# Unambiguous detection of SARS-CoV-2 subgenomic mRNAs with single-cell RNA sequencing

**DOI:** 10.1128/spectrum.00776-23

**Published:** 2023-09-07

**Authors:** Phillip Cohen, Emma J. DeGrace, Oded Danziger, Roosheel S. Patel, Erika A. Barrall, Tesia Bobrowski, Thomas Kehrer, Anastija Cupic, Lisa Miorin, Adolfo García-Sastre, Brad R. Rosenberg

**Affiliations:** 1 Department of Microbiology, Icahn School of Medicine at Mount Sinai, New York, New York, USA; Karolinska Institutet, Huddinge, Sweden

**Keywords:** coronavirus, virus-host interactions, molecular methods, single-cell RNA-seq, transcriptomics, virology, viral pathogenesis

## Abstract

Single-cell RNA sequencing (scRNA-Seq) studies have provided critical insight into the pathogenesis of severe acute respiratory syndrome coronavirus 2 (SARS-CoV-2), the causative agent of coronavirus disease 2019 (COVID-19). scRNA-Seq library preparation methods and data processing workflows are generally designed for the detection and quantification of eukaryotic host mRNAs and not viral RNAs. Here, we compare different scRNA-Seq library preparation methods for their ability to quantify and detect SARS-CoV-2 RNAs with a focus on subgenomic mRNAs (sgmRNAs). We show that compared to 10X Genomics Chromium Next GEM Single Cell 3′ (10X 3′) libraries or 10X Genomics Chromium Next GEM Single Cell V(D)J (10X 5′) libraries sequenced with standard read configurations, 10X 5′ libraries sequenced with an extended length read 1 (R1) that covers both cell barcode and transcript sequence (termed “10X 5′ with extended R1”) increase the number of unambiguous reads spanning leader-sgmRNA junction sites. We further present a data processing workflow, single-cell coronavirus sequencing (scCoVseq), which quantifies reads unambiguously assigned to viral sgmRNAs or viral genomic RNA (gRNA). We find that combining 10X 5′ with extended R1 library preparation/sequencing and scCoVseq data processing maximizes the number of viral UMIs per cell quantified by scRNA-Seq. Corresponding sgmRNA expression levels are highly correlated with expression in matched bulk RNA-Seq data sets quantified with established tools for SARS-CoV-2 analysis. Using this scRNA-Seq approach, we find that SARS-CoV-2 gene expression is highly correlated across individual infected cells, which suggests that the proportion of viral sgmRNAs remains generally consistent throughout infection. Taken together, these results and corresponding data processing workflow enable robust quantification of coronavirus sgmRNA expression at single-cell resolution, thereby supporting high-resolution studies of viral RNA processes in individual cells.

**IMPORTANCE:**

Single-cell RNA sequencing (scRNA-Seq) has emerged as a valuable tool to study host-virus interactions, especially for coronavirus disease 2019 (COVID-19). Here we compare the performance of different scRNA-Seq library preparation methods and sequencing strategies to detect SARS-CoV-2 RNAs and develop a data processing workflow to quantify unambiguous sequence reads derived from SARS-CoV-2 genomic RNA and subgenomic mRNAs. After establishing a workflow that maximizes the detection of SARS-CoV-2 subgenomic mRNAs, we explore patterns of SARS-CoV-2 gene expression across cells with variable levels of total viral RNA, assess host gene expression differences between infected and bystander cells, and identify non-canonical and lowly abundant SARS-CoV-2 RNAs. The sequencing and data processing strategies developed here can enhance studies of coronavirus RNA biology at single-cell resolution and thereby contribute to our understanding of viral pathogenesis.

## INTRODUCTION

Severe acute respiratory syndrome coronavirus 2 (SARS-CoV-2) is the causative agent of coronavirus disease 2019 (COVID-19), which as of January 2023 has caused over 663 million cases and over 6.7 million deaths globally (1, 2). Global efforts to understand the pathogenesis of SARS-CoV-2 infection have led to the development of vaccines and antiviral drugs, which have significantly reduced morbidity and mortality (3). “Omics” methods have been instrumental in studying SARS-CoV-2 in part because they have generated large amounts of data regarding host-viral interactions at unprecedented speed (4 – 15). Single-cell RNA sequencing (scRNA-Seq) studies in particular have been used to study multiple aspects of SARS-CoV-2 infection including but not limited to viral tropism (16 – 22), peripheral immune changes (23 – 33), transcriptional changes induced by infection (34, 35), and to develop cell atlases of COVID-19 pathology (23, 24, 36, 37). Of note, most scRNA-Seq workflows have been developed and optimized for studies of eukaryotic transcription but not viral, specifically SARS-CoV-2, transcription. The performance of different scRNA-Seq library preparation methods, sequencing strategies, and data processing workflows to detect and quantify viral RNAs may impact the analysis and interpretation of such studies.

SARS-CoV-2 is a betacoronavirus with a 29 kB positive-sense, single-stranded RNA genome (38, 39). SARS-CoV-2 generates genomic RNA (gRNA), subgenomic mRNAs (sgmRNAs), and negative-sense antigenomic RNA during active infection (40, 41). Both gRNA and sgmRNAs are polyadenylated, which enables detection by scRNA-Seq protocols that rely on poly-T primed reverse transcription (39 – 41). Translation of gRNA results in the production of one of two polyproteins, pp1a and pp1ab, which are subsequently cleaved into an array of non-structural proteins involved in pathogenesis and replication (39, 41). Translation of sgmRNAs generates structural and accessory viral proteins critical for virion production and pathogenesis (39, 41). Specific detection of sgmRNAs is therefore necessary for the analysis of viral gene expression dynamics within and across cells and viruses.

**Fig 1:**
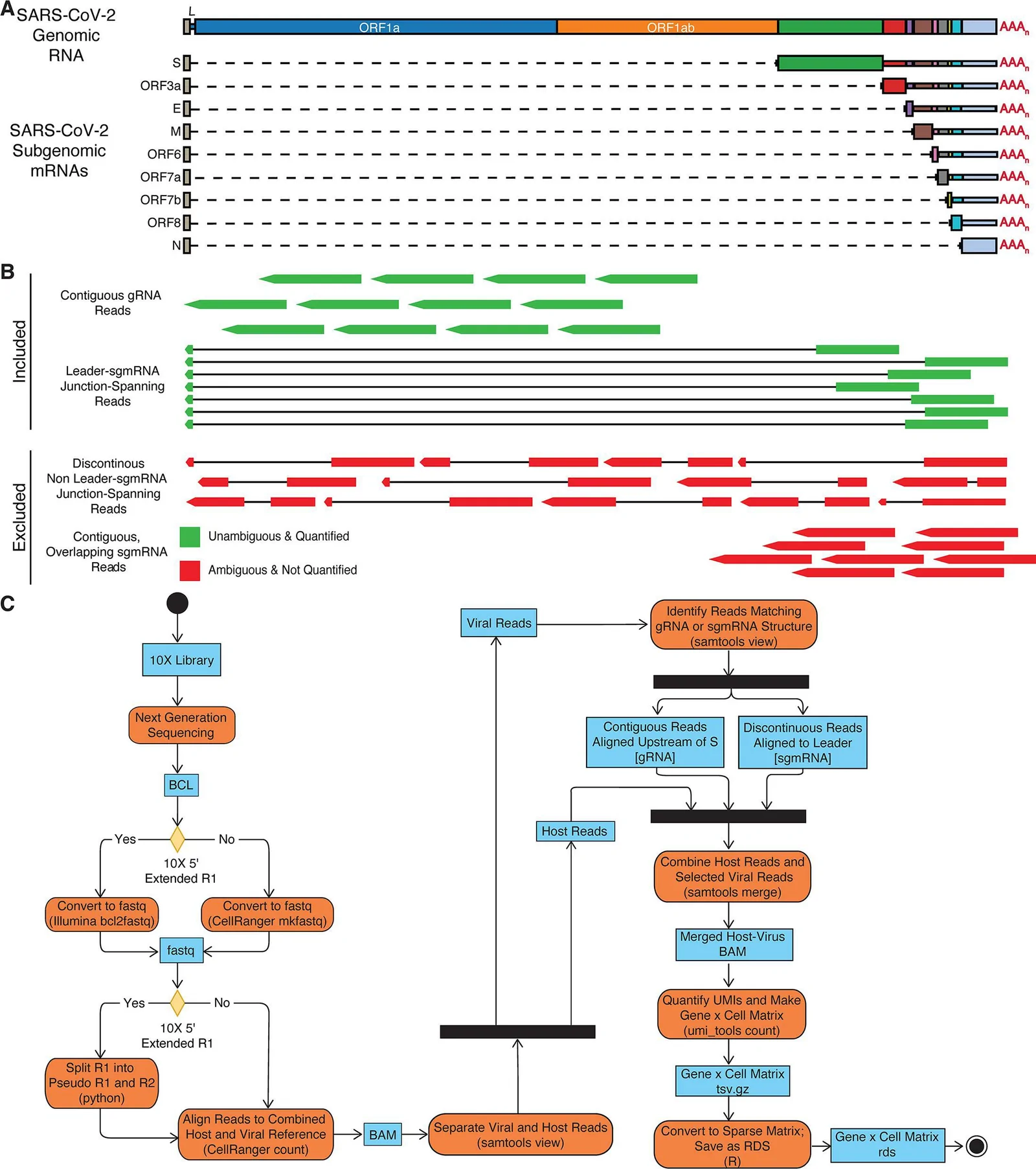
(A) Illustration of SARS-CoV-2 genomic RNA, gRNA, and sgmRNAs. (B) *Top:* Reads included for analysis by scCoVseq. Either (1) contiguous reads mapping to ORF1a/b and therefore derived from gRNA or (2) discontinuous reads spanning the leader region and ORFs transcribed by sgmRNAs. *Bottom*: Reads excluded from analysis by scCoVseq. Either (1) discontinuous reads that do not include sequence mapping to the leader region or (2) contiguous reads that map to ORFs other than ORF1a/b, which are ambiguous. (C) Activity diagram of scCoVseq pipeline. Blue rectangles indicate inputs/outputs for each stage. Orange rounded rectangles indicate a process in bold with software indicated.

sgmRNAs are generated by discontinuous transcription events during negative-strand synthesis (40). Transcription regulatory sequences (TRS), present in the 5′ leader sequence of the virus (TRS-L) and upstream of each open reading frame (ORF) body (TRS-B), regulate this process (40). Template switching of the viral polymerase from a TRS-B to a TRS-L generates sgmRNAs with the 5′ leader sequence fused to the sgmRNA ORF body (Fig. 1A) (40). These “nested” sgmRNAs share the viral ORF sequence downstream of the junction site in addition to a common leader sequence upstream of the junction site (40). This redundancy poses a challenge for standard scRNA-Seq data processing pipelines because reads mapping to redundant sgmRNA sequences are categorized as “ambiguous” and typically excluded from quantification. This problem has been addressed in bulk RNA-Seq by quantifying SARS-CoV-2 reads spanning leader-ORF junctions, which unambiguously identify sgmRNAs (12, 15). However, many scRNA-Seq library preparation methods and/or sequencing strategies do not include this region of sgmRNAs at significant coverage due to differences in library format and configuration of sequencing reads. These limitations hinder the high-throughput study of coronavirus sgmRNA production, dynamics, and impact on host gene expression at single-cell resolution.

To address these challenges with the aim of improving detection and quantification of coronavirus sgmRNAs by scRNA-Seq, we examined the impact of both experimental (i.e., scRNA-Seq library preparation method and sequencing strategies) and data processing elements on the detection, resolution, and quantification of SARS-CoV-2 RNA species as captured by the 10X Genomics Chromium scRNA-Seq platform. We found that unambiguous detection of different SARS-CoV-2 RNAs varied widely across 10X Genomics Chromium scRNA-Seq library preparation methods, with increased unambiguous detection of SARS-CoV-2 sgmRNAs with 10X Chromium Next GEM Single Cell V(D)J (10X 5′) compared to 10X Genomics Chromium Next GEM Single Cell 3′ (10X 3′) gene expression library preparation methods. Using a customized sequencing strategy on 10X 5′ libraries, which we call 10X 5′ with an extended read 1 (R1) sequencing, we could further increase the number of unambiguous SARS-CoV-2 reads and resultant quantification of unique molecular identifiers (UMIs) per SARS-CoV-2 RNA. We also developed a data processing workflow, single-cell coronavirus sequencing (scCoVseq), to quantify only unambiguous SARS-CoV-2 sgmRNA reads in scRNA-Seq data. This read assignment workflow yielded similar sgmRNA quantities in aggregated scRNA-Seq data as measured in corresponding bulk RNA-Seq data processed with previously established SARS-CoV-2 analysis tools, supporting its use for scRNA-Seq data sets. Finally, using scRNA-Seq with scCoV-Seq data processing, we show that expression of each viral sgmRNA is highly correlated across individual infected cells, which may suggest that the relative proportion of viral sgmRNA expression is maintained throughout infection.

## MATERIALS AND METHODS

### Cell lines and viral infection

Vero E6 cells (ATCC, CRL-1586) were maintained in Dulbecco’s Modified Eagle Medium (DMEM, Corning) supplemented with 10% fetal bovine serum (FBS) and 1% Penicillin-Streptomycin (PSN, Fisher Scientific), and routinely cultured at 37°C with 5% CO_2_. A549-ACE2 [previously described in references (42, 43)] were maintained in DMEM (Corning) supplemented with 10% FBS (Peak Serum) and PSN at 37°C and 5% CO2. All cell lines used in this study were regularly screened for Mycoplasma contamination with the MycoAlert Detection Kit (Lonza).

All SARS-CoV-2 propagations and experiments were performed in a Biosafety Level 3 facility in compliance with institutional protocols and federal guidelines. For experiments involving Vero E6 cells, SARS-CoV-2 (isolate USA-WA1/2020, BEI resource NR-52281) and control media (mock infected) stocks were grown by inoculating a confluent T175 flask of Vero E6 cells (passage 2). Mock and SARS-CoV-2-infected cultures were maintained in reduced serum DMEM (2% FBS) for 72 h, after which culture media was collected and filtered by centrifugation (8,000× *g*, 15 min) using an Amicon Ultra-15 filter unit with a 100-kDa cutoff filter (Millipore # UFC910024). Concentrated stocks in reduced-serum media (2% FBS), supplemented with 50 mM N-2-hydroxyethylpiperazine-N-2-ethane sulfonic acid (HEPES) buffer (Gibco), were stored at −80°C. Viral titers were determined by plaque assay as previously described (42).

For experiments involving A549-ACE2 cells, a recombinant SARS-CoV-2 (rSARS-CoV-2) virus, rSARS-CoV-2 ORF6 M58R, was used as described elsewhere (44). All viral stocks were grown in Vero E6 cells as previously described and validated by genome sequencing (35). Viral genome sequencing was either performed using the MinION sequencer (Oxford Nanopore Technologies) or with the Nextera XT DNA Sample Preparation kit (Illumina) as described elsewhere (45, 46).

### scRNA-Seq

For scRNA-Seq experiments with Vero E6 cells, cultures in six-well plates were infected with SARS-CoV-2 at a multiplicity of infection (MOI) of 0.1, or with an equivalent volume of control media, in reduced-serum media (2% FBS) for 24 h. To prepare cells for scRNA-Seq, mock and SARS-CoV-2-infected cultures were washed with calcium/magnesium-free PBS and dissociated with TrypLE (Gibco, 5 min at 37°C), after which samples were centrifuged (200× *g*, 5 min), resuspended in calcium/magnesium-free PBS supplemented with 1% BSA, and counted. Mock and SARS-CoV-2-infected cell culture samples were filtered through a 40-µM FlowMi strainer (ScienceWare) and counted prior to loading on the 10X Genomics Chromium Controller according to the manufacturer’s protocol. Mock and infected samples were loaded on separate lanes of a 10X Genomics Chromium Controller for either NextGEM Single Cell 3′ v3.1 (10X 3′) or NextGEM Single Cell V(D)J v1.1 (10X 5′), gene expression library preparation.

scRNA-Seq gene expression libraries were prepared by 10X 3′ and 5′ library construction methods according to the manufacturer’s guidelines. Per 10X Genomics instructions, final library sample index PCR cycle parameters were selected based on quantification and quality assessment (Agilent Bioanalyzer 2100) of intermediate barcoded cDNA samples: 10X 3′ mock and infected gene expression libraries and the 10X 5′ infected gene expression library were PCR amplified for 16 cycles while the 10X 5′ mock gene expression library was amplified for 14 cycles. 10X 3′ gene expression libraries were pooled and sequenced by short-read sequencing on an Illumina NextSeq 500 using a high output 150 cycle reaction kit according to the manufacturer’s protocol with the following read lengths: read 1, 28 nt; i7 index 8 nt; and read 2, 130 nt. 10X 5′ gene expression libraries were also pooled and sequenced with recommended read lengths (read 1, 26 nt; i7 index, 8 nt; and read 2, 132 nt) or with extended R1 protocol (read 1, 158 nt; i7 index 8 nt; no read 2).

For scRNA-Seq experiments with A549 cells stably expressing the SARS-CoV-2 receptor, ACE2 (A549-ACE2), cultures were incubated with virus at an MOI of 2 in DMEM (Corning) supplemented with 2% FBS (Peak Serum), 1% non-essential amino acids (Gibco), 1% HEPES (Gibco), and 1% PSN at 37°C and 5% CO2. As A549-ACE2 cells infected with attenuated rSARS-CoV-2 ORF6 M58R virus exhibit considerably less viral cytopathogenicity than Vero E6 cells infected with SARS-CoV-2 USA-WA1/2020 (44), we were able to use a higher MOI (2) in these experiments to achieve a high proportion of infected cells without excessive cell death. Cells were incubated for 4 h before supernatants were removed and replenished with fresh media. At 24 h post-infection, cells were detached with TrypLE (Gibco), washed with PBS, and passed through a 70-µM filter. Finally, samples were processed using the Next GEM Single Cell Immune Profiling Assay (5´ RNA) single index kit (10X Genomics) according to the manufacturer’s instructions and as described previously, using 12 cycles of amplification during the cDNA amplification step (35). Sequencing was performed by the Icahn School of Medicine at Mount Sinai Genomics Core Facility using an Illumina NovaSeq with read lengths set to 200 bp for read 1, 8 bp for index 1, and 100 bp for read 2. Only read 1 was used for data denoted as 10X 5´ extended R1, and for analyses involving 10X 5' data (i.e., not “extended”) R1 was truncated to 26 bases, the length required for cell barcode and UMI quantification, and read 2 was used without modification.

### scRNA-seq data pre-processing

#### *Conversion of Illumina BCL files to* FASTQ

FASTQ files for standard sequencing of 3′ and 5′ gene expression libraries were generated using the mkfastq command in cellranger v.3.1.0 (10X Genomics). FASTQ files for 5′ libraries sequenced with the extended R1 strategy were generated using bcl2fastq v2.20.0 (Illumina, Inc.). Extended R1 FASTQ files were then separated into “pseudo R1” FASTQ files, containing the cell barcode and UMI, and “pseudo read 2 (R2)” FASTQ files, containing cDNA sequence, using a customized Python (v3.7.3) script (available at GitHub https://github.com/BradRosenbergLab/scCoVseq) as follows. The cell barcode and UMI are selected from the first 26 bp of R1. The subsequent 13 bp from the template switch oligonucleotide and are ignored. The remaining nucleotides (and corresponding quality scores) are reverse complemented and stored as pseudo R2. The read header of the pseudo R2 FASTQ file is modified to reflect the format for standard R2 FASTQ.

#### Downsampling FASTQ files to control for sequencing depth

To control for differences in sequencing depth for each Vero E6 library, read depth per library was downsampled to approximately 50,000 reads per cell. To generate a whitelist of cell barcodes for downsampling while accounting for transcriptional shutdown in SARS-CoV-2-infected cells (35), we generated preliminary gene × cell matrices using cellranger count (v3.1.0, 10X Genomics, Inc.) to quantify and align reads to a host reference (African Green Monkey, ChlSab1.1) combined with SARS-CoV-2 transcripts as annotated by NCBI SARS-CoV-2 reference (NC_045512.2) with modifications for USA/WA01 strain for each data set (modified SARS-CoV-2 reference genome and annotations available at GitHub https://github.com/BradRosenbergLab/scCoVseq). The resulting output was analyzed in R (v4.0.4) with Seurat (v4.0.1) (47 – 49) to filter out putative doublets and empty droplets according to total UMIs/cell, number of genes/cell, and percent of mitochondrial gene expression. Due to increases in apparent background RNA levels in infection samples, as well as minor differences in sequencing saturation across sequencing methods, following data exploration and quality assessment, filtering thresholds were determined independently for each sample (Table S1). After filtering, putative cell-containing cell barcodes were output to a whitelist per library. Based on these whitelists, the initial FASTQ files were downsampled using seqtk (v1.2) (50) to a total read depth of 50,000 multiplied by the number of cells in the library.

#### Preparation of empirically derived SARS-CoV-2 genome reference

Downsampled (for Vero E6 samples) and non-downsampled (for A549-ACE2 samples) FASTQ files were then mapped using cellranger count (v3.1.0, 10X Genomics, Inc.) to an empirically defined reference of SARS-CoV-2 sgmRNAs derived from previously reported SARS-CoV-2 (BetaCoV/Korea/KCDC03/2020) RNAs sequenced with long-read direct RNA Nanopore sequencing (12). These were downloaded from the UCSC Genome Browser Table Browser (51) after filtering for TRS-dependent transcripts and scores >900 and exporting to GTF format. Transcripts for previously unknown ORFs were excluded from the annotation. An additional annotation for “genomic RNA” was included, which covered the entire length of the SARS-CoV-2 genome. Aligning the BetaCoV/Korea/KCDC03/2020 genome with USA/WA-CDC-WA1/2020 genome showed that the USA/WA-CDC-WA1/2020 reference had an additional 21 3′ adenosine nucleotides annotated. To account for this in our reference, we extended any annotations from BetaCoV/Korea/KCDC03/2020 that ended at the 3′ end of the genome by an additional 21 bases. This SARS-CoV-2 reference was appended to the host ChlSab1.1 Ensembl reference for Vero E6 analyses or GRCh38 (version 100) human reference for ACE2-A549 analyses.

### scCoVseq

To unambiguously assign and quantify scRNA-Seq reads to SARS-CoV-2 RNAs, the cellranger output BAM was filtered for reads mapping to SARS-CoV-2 or host references using samtools (v1.11) (52). SARS-CoV-2 aligned reads were then subset to putative genomic RNA reads or sgmRNA reads as follows. Genomic reads were defined as those containing no gaps in their alignment and mapping upstream of the start of the most 5′ sgmRNA, S. sgmRNA reads were defined as SARS-CoV-2 reads containing a gap and mapping in part to the 5′ leader sequence, defined as the 5′ proximal 80 nucleotides of the SARS-CoV-2 genome, and in part 3′ to the start of S. All other reads mapping to the SARS-CoV-2 genome were discarded. Reads passing these filtering steps were quantified with umi_tools (v1.0.0) (53). An R (v3.5.3) script using the Matrix (v1.2–18) (54) and readr (v1.3.1) (55) packages was used to convert this to a sparse matrix and save it as an RDS file. UMIs assigned to multiple genes were removed from the resulting matrix during downstream analysis.

### scRNA-seq data analysis

#### Sashimi plots

Reads from Vero E6 samples for 10X 3′, 10X 5′, and 10X 5′ extended R1 data that aligned to the SARS-CoV-2 reference by cellranger were subset from the cellranger output BAM file. Each BAM file was downsampled to approximately 1 × 10^6^ reads to control for differences in sequencing depth across libraries. Sashimi plots were generated with ggsashimi (v1.0.0) (56).

#### Classification of SARS-CoV-2-infected cells

scCoVseq-derived gene by cell matrices was loaded into R (v4.0.4) and analyzed with the Seurat (v4.0.1) (47 – 49) package. For each 10X method, mock and infected gene × cell matrices were merged with the Seurat merge command. To identify infected and bystander cells within SARS-CoV-2-treated cultures, the Euclidean distance between the z-scaled expression of SARS-CoV-2 sgmRNA UMIs per cell was clustered using k-medoids by the pam algorithm (*k* = 2) implemented in the cluster (v2.1.2) package (57). Output clusters were then compared for viral UMI expression per cell, and the cluster with more viral UMIs was classified as infected and the other as uninfected.

#### Comparison of SARS-CoV-2 RNA UMIs per scRNA-seq method

To examine the distribution of SARS-CoV-2 UMIs per cell by scRNA-Seq library preparation method/sequencing strategy, the 25th percentile of total UMIs was quantified for all infected cells from each 10X library preparation method/sequencing strategy. Any cells with fewer UMIs than the minimum 25th percentile of all samples were discarded, and all cells were subsequently downsampled to this same number of total UMIs/cell using the Seurat SampleUMI command. Each data set was randomly downsampled to the same number of infected cells to equalize for differences in cell numbers, and viral sgmRNA UMIs/cell were plotted by library preparation method/sequencing strategy.

#### SARS-CoV-2 read distribution by scRNA-seq library preparation method/sequencing strategy

SARS-CoV-2 reads were defined as genomic or subgenomic using scCoVseq. Reads aligning to the SARS-CoV-2 reference that were excluded from scCoVseq were classified as ambiguous. The number of genomic, subgenomic, or ambiguous reads per million SARS-CoV-2 reads was calculated and plotted for each scRNA-Seq library preparation method/sequencing strategy.

#### Differential expression analysis

To explore expression differences between infected, bystander, and mock cells, differential expression testing with edgeR (v3.32.1) was performed with modifications for scRNA-Seq as previously described (58, 59). Viral genes were excluded from the analysis. To minimize false detection of differential expression for low expression and/or minimally detected transcripts, host genes detected in fewer than 10% of cells in each condition (mock, infected, and bystander) were excluded. To account for differences in the RNA content of infected cells due to virus-induced transcriptional shutdown, all cells were downsampled to the 25th percentile of total UMIs of infected cells. Cells with fewer UMIs than the threshold were excluded from the analysis. Differential gene expression was performed with edgeR using a generalized linear model quasi-likelihood F test adapted with a term for gene detection rate (58, 59). Genes with an absolute log_2_ fold change greater than or equal to 1 and a false discovery rate less than 0.05 were considered significant. For KEGG enrichment analysis, pairwise tests between mock, bystander, and infected cells were performed. Differentially expressed genes with an absolute log_2_ fold change greater than or equal to 1 and a false discovery rate less than 0.05 were considered significant and subject to KEGG enrichment analysis using the KEGG annotations for African Green Monkey as implemented in the edgeR function kegga.

#### Quantification of SARS-CoV-2 sgmRNA junction sites

We explored the ability of our extended R1 sequencing strategy to detect SARS-CoV-2 sgmRNA junctions using STARsolo (version 2.7.8a) (60). Aligned reads were re-mapped to the empirical SARS-CoV-2 annotation described above and junction sites per cell were quantified. The resulting junction per cell matrix was plotted in R (v4.0.4).

### Bulk RNA-seq

#### Bulk RNA-seq library preparation and sequencing

A549-ACE2 samples for bulk RNA sequencing were homogenized in Trizol Reagent (Invitrogen) and total RNA was extracted using the miRNeasy mini kit (Qiagen) per the manufacturer’s instructions. DNAse treatment was performed on isolated RNA using the RNA Clean and Concentrator Kit (Zymo). Total RNA was examined for quantity and quality using the TapeStation (Agilent) and Quant-It RNA (ThermoFisher) systems. RNA samples with sufficient material (10 pg–10 ng) were passed to whole-transcriptome library preparation using the SMART-Seq v4 PLUS Kit (Takara Bio) following the manufacturer’s instructions. This library construction method was selected due to molecular similarities (5′ template switch adaptor addition, full-length cDNA amplification, etc.) to 10X Genomics Chromium scRNA-Seq library preparation. Briefly, total RNA inputs were normalized to 10 ng in 10.5 µL going into preparation. 3′ ends of cDNA were then adenylated prior to ligation with adapters utilizing unique dual indices (96 UDIs) to barcode samples to allow for efficient pooling and high-throughput sequencing. Libraries were enriched with PCR, with all samples undergoing 14 cycles of amplification prior to purification and pooling for sequencing. Bulk RNA sequencing was conducted on dual index libraries using a 300 cycle Mid Output kit on an Illumina NextSeq 500 with standard read configurations for R1, i7 index, i5 index, and R2: 150 nt, 8 nt, 8 nt, 150 nt.

#### Bulk RNA-seq analysis

Raw BCL files were converted to FASTQ files using bcl2fastq (v2.20.0, Illumina, Inc.). For quantification of SARS-CoV-2 sgRNA and gRNA expression, the periscope (v0.1.2) package was used with the technology argument set to “illumina" (61). Finally, sgRNA reads per total mapped reads were calculated.

### Flow cytometry

Vero E6 cells were dissociated, fixed with 4% paraformaldehyde at room temperature for a minimum of 24 h, washed once with PBS and permeabilized with 1× perm-wash buffer (BD Biosciences) for 5 min. SARS-CoV nucleocapsid (N) antibody (clone 1C7C7) (kindly provided by Thomas Moran, Icahn School of Medicine at Mount Sinai, New York, NY) conjugated to AlexaFluor 647 was diluted 1:400 in perm-wash buffer, and added directly to samples. Samples were then incubated at room temperature for 40 min in the dark. After staining, samples were washed once with 1× perm-wash buffer, once with PBS, resuspended in FACS buffer (PBS supplemented with 1% FBS), and acquired on a Gallios flow cytometer (Beckman-Coulter). For all viral infections, analysis was performed with FlowJo software (v10.7.1, Becton Dickinson), excluding cell doublets and debris and gating according to mock-infected populations.

### Immunofluorescence microscopy

Vero E6 cells were seeded in six-well plates (Falcon) with one glass coverslip (Fisher Scientific) per well. At 24 h post-infection, cells were washed with PBS and fixed with 4% paraformaldehyde (Fisher Scientific) overnight. Fixed cells were permeabilized using 0.1% Triton-X (Fisher Scientific) in PBS and blocked with 4% bovine serum albumin (BSA, Fisher Scientific) in PBS. Blocked coverslips were incubated with SARS-N-1C7 (1:500 in 4% BSA PBS) overnight at 4C, washed three times with PBS, and incubated for 45 min with 1:500 AlexaFluor 488-conjugated anti-mouse (Invitrogen, 1:500 in 4% BSA PBS) plus DAPI (Thermo Fisher Scientific, 1:1,000 in 4% BSA PBS) at room temperature. Coverslips were stained with phalloidin (1:400 in PBS) for 1 h at room temperature and washed again three times with PBS. Coverslips were mounted using Prolong Diamond (Life Technologies P36970). Confocal laser scanning microscopy was performed using a Leica SP5 DMI (ISMMS Microscopy CoRE and Advanced Bioimaging Center) with a 40×/1.25 oil objective. Images were collected at a resolution of 512 × 512 pixels in triplicate per slide. Images were processed and analyzed using LAS X and CellProfiler (v4) (62).

## RESULTS

SARS-CoV-2 generates gRNA and sgmRNAs during infection, which are highly redundant in their sequences (Fig. 1A). In scRNA-Seq data processing pipelines, reads mapping to redundant sequences are “assigned” to all genes containing that sequence and are typically excluded from quantification steps as ambiguous. We therefore identified read structures that could unambiguously identify gRNA or different species of sgmRNAs to allow for their specific quantification (Fig. 1B). Reads derived from gRNA should be contiguous and could map anywhere on the SARS-CoV-2 genome. Reads derived from sgmRNA could be either gapped or contiguous and could map to the 5′ leader and/or downstream of the start site of S, the most 5′ sgmRNA. Because contiguous reads mapping downstream of S could derive either from gRNA or from sgmRNAs, they were excluded from quantification. We therefore defined gRNA reads as contiguous reads aligning upstream of regions contained in sgmRNAs. sgmRNA reads were defined as discontinuous reads spanning the leader region and regions used by sgmRNAs. Reads that did not match either of these formats could not be unambiguously assigned to gRNA or a sgmRNA and were therefore excluded from quantification (Fig. 1B). With this framework, we developed the scCoVseq data processing workflow to quantify unambiguous genomic and subgenomic viral reads from scRNA-Seq data (Fig. 1C).

**Fig 2:**
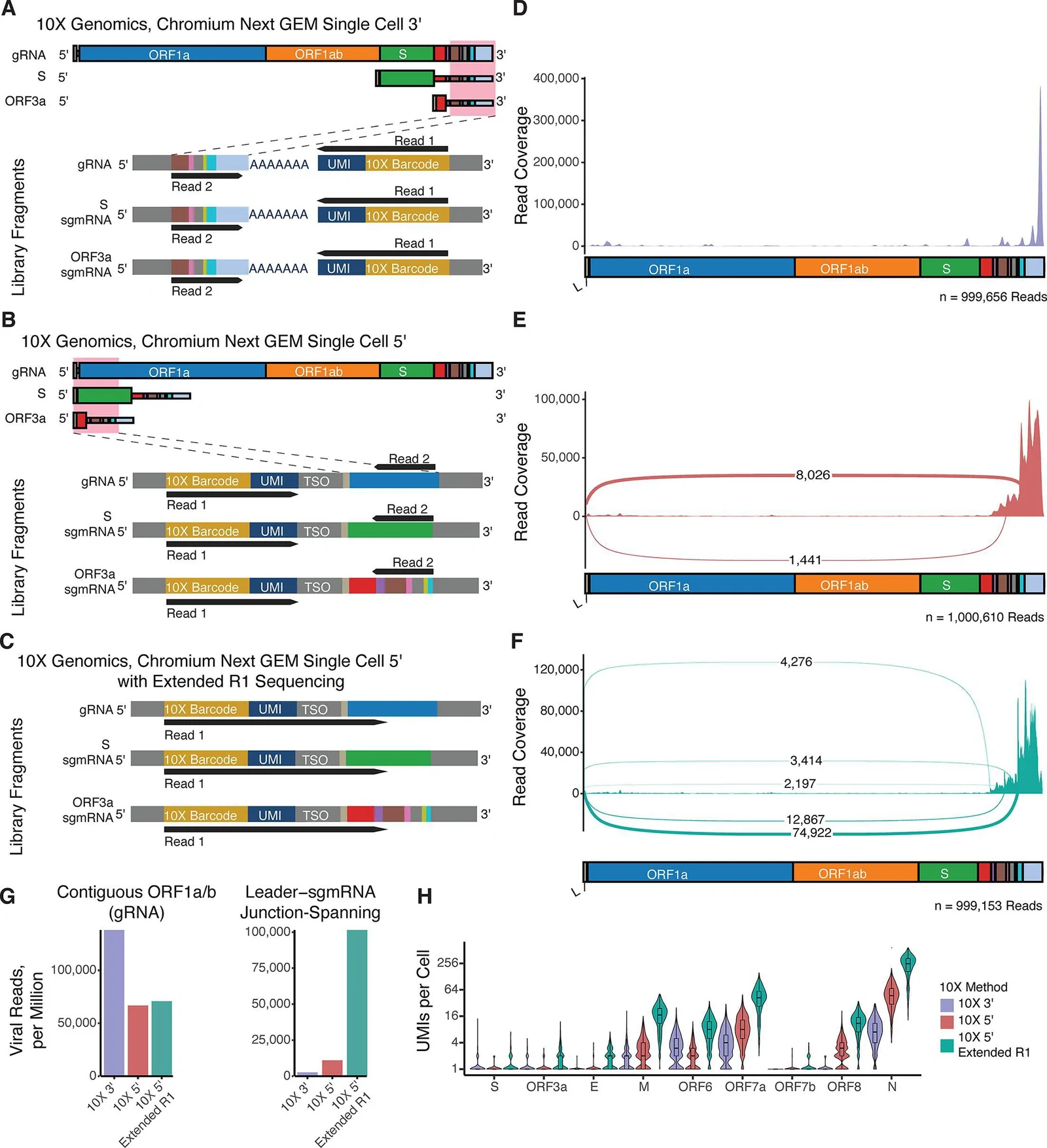
(A–C) Illustration of gRNA and S and ORF3a sgmRNAs. A red box indicates regions contained in the final 10X library. *Lower:* Example illustration of 10X library fragments derived from gRNA and S and ORF3a sgmRNAs with sequencing read 1 and read 2 indicated. 10X 3′ (A), 10X 5′ (B), and 10X 5′ extended R1 (C) libraries are illustrated. (D–F) Sashimi plot of 10X 3′ (D), 10X 5′ (E), and 10X 5′ extended R1 (F) reads mapped to the SARS-CoV-2 genome filtered to show only junctions supported by at least 1,000 reads. Total number of reads visualized is indicated in the bottom right. (G) Reads per million reads mapped to SARS-CoV-2 reference in 10X 3′, 10X 5′, or 10X 5′ with extended R1 data. (H) UMIs per cell for all sgmRNAs in infected cells in each data set. Each data set was downsampled to an equal number of infected cells and each cell’s total UMIs were downsampled to the same value to account for differences in sequencing depth. The leader region is enlarged (*not to scale*) in illustrations of the genome for visibility. All data in this figure are derived from SARS-CoV-2 (USA-WA1/2020)-infected Vero E6 cells. L = Leader.

We first used scCoVseq to compare the abilities of different scRNA-Seq library preparation methods and sequencing strategies to quantify SARS-CoV-2 RNAs in Vero E6 cell line infection. Vero E6 is an African Green Monkey (*Chlorocebus sabaeus*) epithelial cell line that does not produce interferons in response to viral infection (63 – 65); it is widely used for SARS-CoV-2 propagation and molecular studies for its support of robust viral replication and production of high viral titers. In the frequently employed Chromium scRNA-Seq system developed by 10X Genomics, Inc., there are two library preparation methods for droplet-based scRNA-Seq: 10X 3′ and 10X 5′. 10X 3′ generates library fragments derived from the 3′ regions of polyadenylated RNAs within a cell (Fig. 2A). Because sgmRNAs share all viral sequence 3′ of the leader-body junction site, 10X 3′ library fragments derived from SARS-CoV-2 predominantly align to the 3′ end of the viral genome and do not contain leader-ORF junctions (Fig. 2D). These reads are limited in their ability to differentiate gRNA from sgmRNA or distinguish different sgmRNA species. 10X 5′ generates library fragments from the 5′ termini of polyadenylated RNAs (Fig. 2B). These fragments are on average approximately 500 bp long (according to the manufacturer’s documentation) and would be expected to contain leader-ORF junctions of SARS-CoV-2 sgmRNAs. The transcript read (R2), however, derives from the 3′ end of these fragments and at the recommended read length of 91 bases is not long enough to consistently sequence into the leader-sgmRNA junction site (Fig. 2B). Reads from 10X 5′ can therefore contain some but not all junctions (Fig. 2E). We reasoned that we could use 10X 5′ library fragments to detect junction-spanning reads by sequencing from the 5′ end of the fragment. To do this, we used an alternative sequencing strategy in which we extended R1, which is normally used to sequence the cell barcode and UMI, to sequence into the leader-body junction site (Fig. 2C). Using 10X 5′ with extended R1, we were able to sequence more leader-sgmRNA junction sites and increase our ability to unambiguously quantify sgmRNAs (Fig. 2F). Indeed, 10X 5′ extended R1 increased the number of leader-sgmRNA-spanning reads over 10X 5′ and 10X 3′ (Fig. 2G).

Unexpectedly, we observed a larger number of reads classified as gRNA in 10X 3′ compared to 10X 5′ or 10X 5′ with extended R1 (Fig. 2G), and there appears to be increased read coverage in ORF1a and ORF1ab in the 10X 3′ library as well (Fig. 2D). This phenomenon was also reported by Ravindra et al in their 10X 3′ libraries, who showed that these are non-canonical SARS-CoV-2 transcripts and not artifacts of the 10X 3′ library preparation (21). It is unclear at this time, however, why these transcripts may be better detected with 10X 3′ than with 10X 5′.

When quantified with the scCoVseq data processing workflow, we found that 10X 5′ extended R1 yields more UMIs for each sgmRNA per cell compared to 10X 5′ or 10X 3′ (Fig. 2H). Importantly, the average host gene expression per sample was significantly correlated across 5′ library sequencing strategies, suggesting that host gene measurements were minimally affected by 10X 5′ extended R1 sequencing modifications (Fig. S1A through C). For select host genes, we observed higher detection in 10X 5′ than in 10X 5′ extended R1 in Vero E6 cell data. Interestingly, however, when analyzing data from human A549-ACE2 cells, we did not see any notable differences in the quantification of host genes between 10X 5′ and 10X 5′ extended R1 (Fig. S1D). These differences, which are apparent for a small subset of total genes, may be a consequence of incomplete annotation of the African Green Monkey transcriptome reference, as they are not apparent with data mapped to the more thoroughly curated human reference. Furthermore, in all of these analyses, viral genes were shown to be detected at higher levels (in aggregate) with 10X 5′ extended R1 without impacting host gene quantification. Taken together, 10X 5′ libraries sequenced with extended R1 sequencing result in a greater number of unambiguous reads derived from sgmRNAs over 10X 3′ or 10X 5′, and consequently recover more sgmRNA UMIs/cell without affecting host gene quantification.

**Fig 3:**
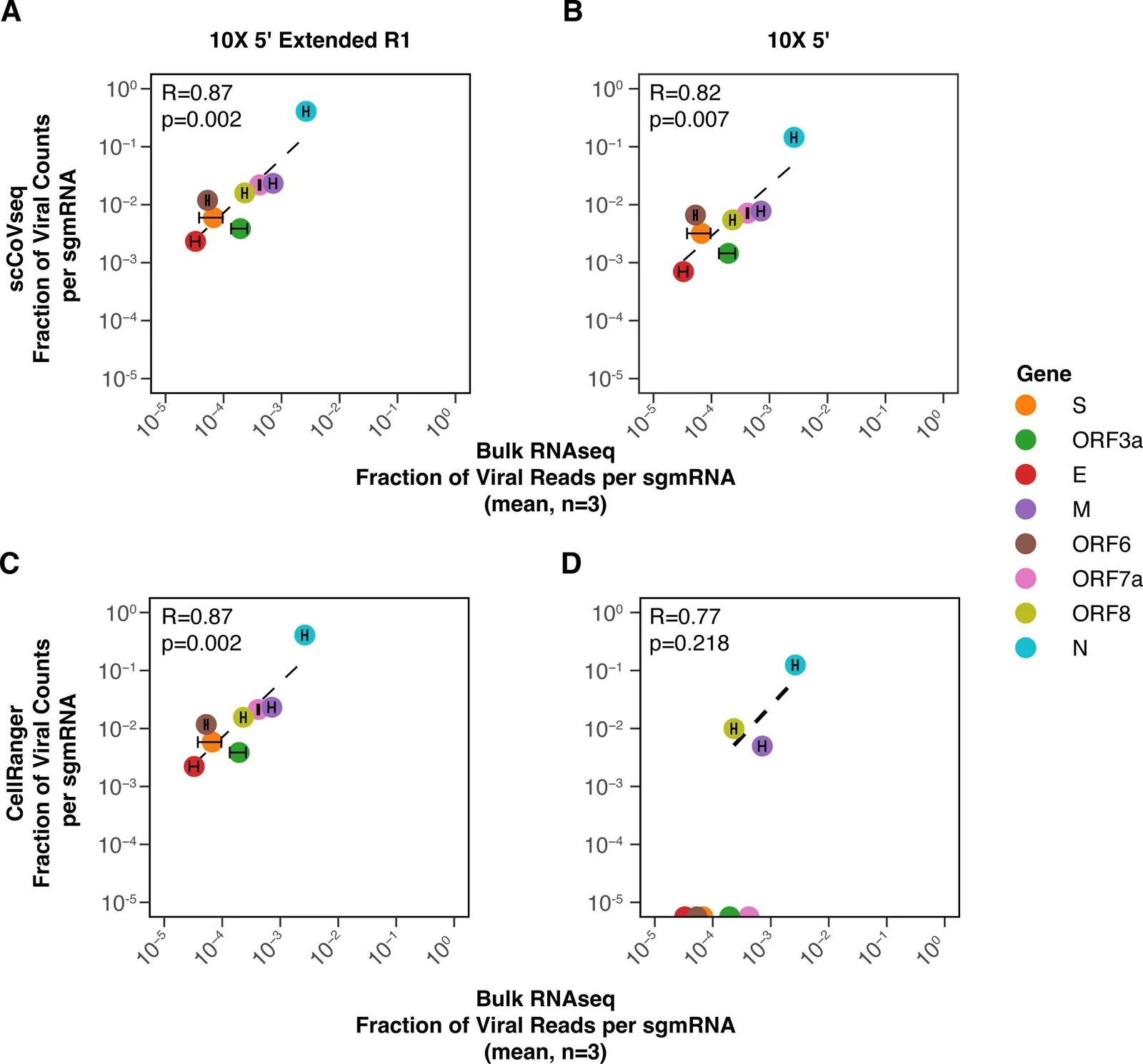
(A–D) Correlation between mean viral sgmRNA quantification in bulk RNA-Seq by periscope and aggregated “pseudobulk” scRNA-Seq data of rSARS-CoV-2 ORF6 M58R-infected ACE2-expressing A549 cells. The mean of three values for the fraction of total viral reads derived from each sgmRNA as quantified in bulk RNA-Seq is shown on the *x*-axis. *x* variable error bars indicate one standard deviation above or below the mean of bulk RNA-Seq sgmRNA fraction. The fraction of total viral UMIs derived from each sgmRNA as quantified in scRNA-Seq is shown on the *y*-axis. Each point indicates a specific ORF. A regression line is fit to each graph, and Pearson’s correlation and resultant *P* values are shown for each analysis. For CellRanger quantified 10X 5′ data in (D), the regression line and Pearson’s correlation do not include S, ORF3a, E, ORF6, or ORF7a due to insufficient detection.

We next explored the ability of the scCoVseq data processing workflow to quantify sgmRNAs as compared to bulk RNA-Seq. For this experiment, we aimed to evaluate scCoVseq in human cells and minimize the often pronounced viral cytopathogenicity observed in Vero E6 cells; therefore, we infected the interferon-competent A549 human lung adenocarcinoma cell line (engineered to express the SARS-CoV-2 receptor, ACE2), with an attenuated mutant virus, recombinant SARS-CoV-2 (rSARS-CoV-2) ORF6 M58R, to allow for higher MOI infection without excessive cell death (44). In parallel to scRNA-Seq library preparation, triplicate bulk RNA-Seq libraries were prepared from these cultures and quantified with periscope, a previously established method to quantify SARS-CoV-2 sgmRNAs in bulk RNA-Seq data using partial alignments of viral reads to TRS and ORF sequence (61). We compared the detection of sgmRNAs in aggregated “pseudobulk” scRNA-Seq data (i.e., summed across all cells) to the mean expression values in the bulk RNA-Seq data; these comparisons to bulk RNA-Seq data were performed for 10X 5′ data sets sequenced with standard or extended R1 strategies, and also for data sets quantified by standard CellRanger or our scCoVseq data processing workflow (Fig. 3). We found that both 10X 5′ extended R1 sequencing strategy (with either quantification scheme) or quantifying 10X 5′ with scCoVseq data processing improved the correlation between aggregated scRNA-Seq data and bulk RNA-Seq data. The combination of 10X 5′ extended R1 sequencing strategy and scCoVseq data processing did not appear to improve further the correlation between scRNA-Seq and bulk RNA-Seq data. Of note, “standard’ CellRanger quantification with “standard” 10X 5′ sequencing resulted in considerably lower expression values than bulk RNA-Seq for all sgmRNAs except M, ORF8 and M. This analysis also showed that, in general, scRNA-Seq tends to detect higher levels of N and ORF6 sgmRNA and lower levels of ORF3a and M sgmRNA than bulk RNA-Seq. This may be due to differences in the properties of single cell as compared to bulk RNA-Seq library preparation or differences in the quantification of reads by the data processing workflows used for each. Overall, these data suggest that 10X 5′ with extended R1 sequencing or scCoVseq data processing improves the correlation between viral sgmRNA and bulk RNA-Seq quantification, but as described above (Fig. 2H), only the combination of both maximizes the number of UMIs detected per sgmRNA per cell.

**Fig 4:**
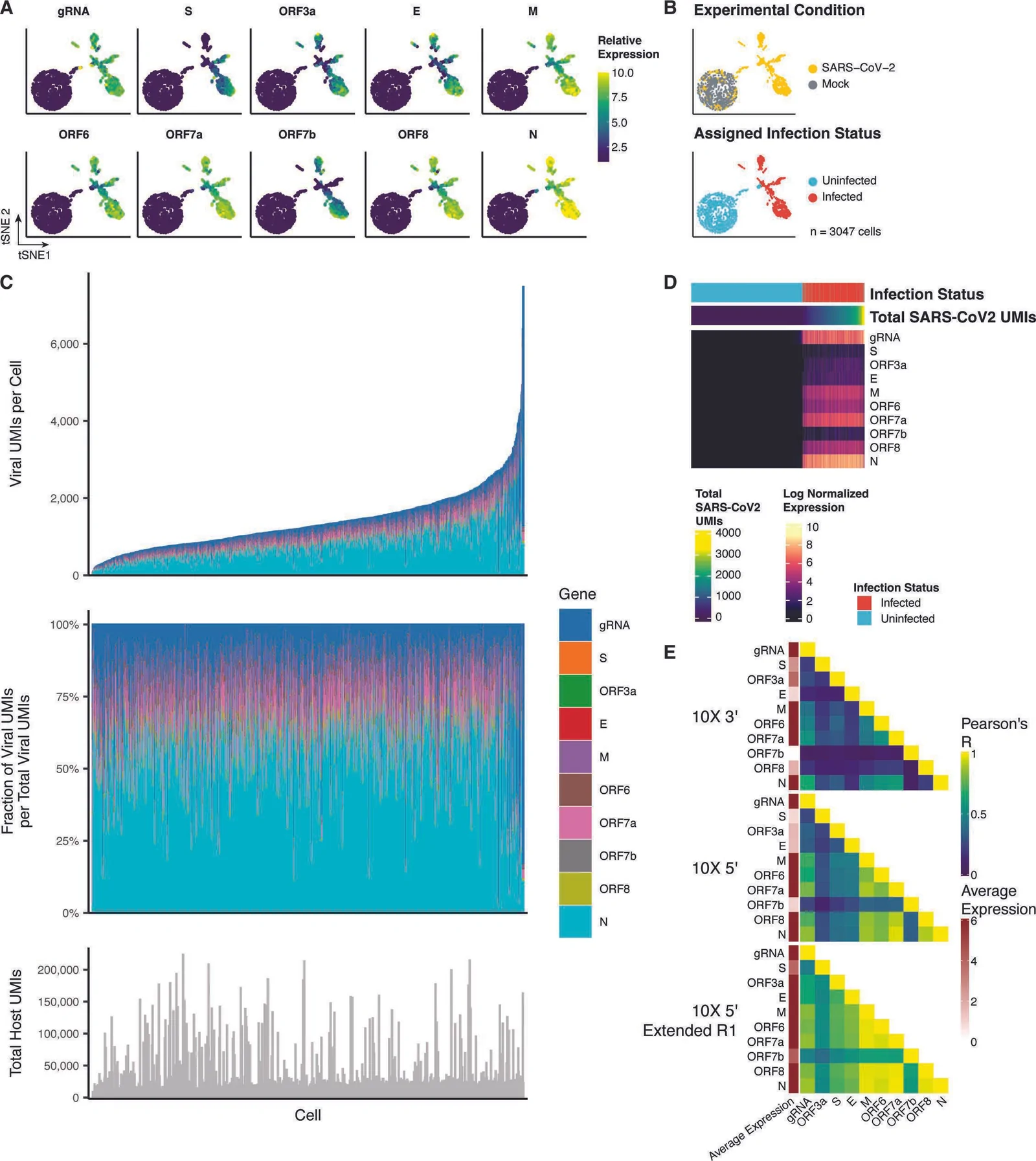
(A) Viral gene expression in *n* = 3,047 mock and infected Vero E6 cells prepared and sequenced by 10X 5′ extended R1 and processed with scCoVSeq. Cells are embedded in tSNE space derived from the Euclidean distance of scaled viral sgmRNA expression (viral gRNA and nine viral sgmRNA genes, as labeled). (B) Experimental condition and assigned infection status of cells, corresponding to tSNE in (A). (C) Top: Total viral UMIs per cell are plotted with each cell representing a single bar and cells ordered from left to right according to the increasing total number of viral UMIs. The number of viral UMIs contributed by each viral gene is indicated by color. Middle: The fraction of viral UMIs derived from each viral gene scaled to 100% is shown with the color of each bar indicating the viral gene. Bottom: The total number of host UMIs detected per cell. (D) Expression of viral genes in infected and uninfected Vero E6 cells. Each column corresponds to a single cell and expression of each viral gene is indicated by color. Cells are ordered left to right by increasing total SARS-CoV-2 viral UMI counts. Cells with total SARS-CoV-2 viral UMIs greater than the 99th percentile are “clipped” to the 99th percentile value for improved visibility. Cells are annotated above with assigned infection status. (E) Pearson’s correlation of log-transformed viral gene expression for each sgmRNA to all other sgmRNAs across individual cells as measured by 10X 3′, 10X 5′, and 10X 5′ extended R1. Sidebar annotation indicates an average sgmRNA expression detected by each sequencing method.

Next, we sought to assess the expression patterns of SARS-CoV-2 sgmRNAs across individual infected cells. We analyzed Vero E6 cells 24 h post-infection with SARS-CoV-2 at an MOI of 0.1 prepared and sequenced with 10X 5′ extended R1 and processed with scCoVseq. We were able to quantify sgmRNAs and gRNA at single-cell resolution (Fig. 4A). We classified infected cells using a k-medoid clustering approach based on sgmRNA expression (Fig. 4B). We found that this classification method effectively separated cells with high levels of viral RNA from cells with low viral RNA (Fig. S2C) and detected a similar percentage of infected cells as detected using flow cytometry and immunofluorescence microscopy of the same cultures (Fig. S3A through C). We observed a progressive increase in all viral RNAs as total viral RNA UMI count increased in infected cells (Fig. 4C). We further found that viral gene expression was highly correlated across cells, suggesting that the relative proportions of viral gene expression are tightly correlated throughout infection with SARS-CoV-2 (Fig. 4D and E). Similar correlations were observed in rSARS-CoV-2 ORF6 M58R-infected ACE2-expressing A549 cells (Fig. S4A and B). Importantly, we found that this pattern was less apparent in library preparation methods and sequencing strategies with suboptimal detection of SARS-CoV-2 RNAs, that is, 10X 3′ and 10X 5′, which further supports the utility of the extended R1 sequencing strategy in studying viral gene expression at single-cell resolution (Fig. 4E).

Using our infection classifications, we performed differential expression testing of infected cells compared to bystander cells within the same Vero E6 culture and to cells in a mock-infected culture. As previously described (35), we observed suppression of many host genes in infected cells accompanied by an upregulation of genes associated with cellular stress response (Fig. S5A and B). We further observed that, while bystander and mock cells had generally similar gene expression patterns, a small number of genes were upregulated in bystander cells compared to mock cells. This is especially notable given the inability of Vero E6 cells to produce interferons in response to viral infection (64). KEGG enrichment analysis of differentially expressed genes in pairwise comparisons of infected, mock, and bystander cells showed that genes previously characterized as “related to COVID-19” were enriched in our infected cells, thereby further supporting the use of single-cell resolution viral sgmRNA expression for infection classification (Fig. S5C).

Finally, we explored the ability of the 10X 5′ extended R1 sequencing strategy to detect non-canonical leader-sgmRNA junctions in single cells. Multiple studies have reported on a variety of non-canonical sgmRNA structures detected in bulk RNA-Seq studies of SARS-CoV-2 infection including TRS-independent sgmRNAs (12, 13), but the physiological significance of these RNAs is currently incompletely understood. We hypothesized that 10X 5′ extended R1 might be able to detect junction sites corresponding to non-canonical sgmRNA structures because of its increased read coverage at expected junction sites. We observed a variety of non-canonical sgmRNA structures including TRS-independent sgmRNAs in our Vero E6 data sequenced with 10X 5′ extended R1 (Fig. S6). While our data were too sparse to compare the specific expression of sgmRNA junctions per individual cell, future studies may be able to leverage 10X 5′ extended R1 to identify differential viral or host gene expression patterns associated with the expression of certain sgmRNA junctions.

## DISCUSSION

In this study, we examined the ability of two commonly used scRNA-Seq library preparation methods, 10X 3′ and 10X 5′, to detect and quantify SARS-CoV-2-derived RNAs with a focus on sgmRNAs. Because of the redundant nature of coronavirus sgmRNA sequences, we developed the scCoVseq data processing workflow, which unambiguously quantifies both sgmRNAs and gRNAs in 10X data. We found that different 10X library preparation methods generate unambiguous leader-sgmRNA junction-spanning reads to different degrees. We were able to increase the detection of leader-sgmRNA junction-spanning reads by extending the length of R1 during the sequencing of 10X 5′ libraries, an approach we term 10X 5′ extended R1 sequencing. We further showed that analyzing 10X 5′ with the scCoVseq data processing workflow or sequencing 10X 5′ with the extended R1 sequencing strategy improved the correlation of scRNA-Seq data with bulk RNA-Seq data. Combining 10X 5′ extended R1 sequencing with scCoVseq data processing maximized quantification of sgmRNA UMIs compared to 10X 3′ or 10X 5′ with standard sequencing. Thus, while processing 10X 5′ with scCoVseq or sequencing with extended R1 improved the confidence of sgmRNA quantification, the combination of 10X 5′ library preparation with extended R1 sequencing and scCoVseq data processing also maximized the number of sgmRNAs detected per cell.

10X 5′ library preparation with extended R1 sequencing and/or scCoVseq data processing enables the comparison of sgmRNA expression across individual cells. We observed that SARS-CoV-2 viral gene expression is highly correlated across cells, and relatedly, that relative proportions of viral RNA species are maintained across increasing levels of total viral RNA. This differs significantly from other viruses such as influenza (66, 67), HSV (68), and CMV (69). These differences are likely due to differences in viral genome structure resulting in differences in the regulation of viral gene expression. For example, the segmented nature of influenza virus genomes can result in virions missing gene segments, which partially explains increased heterogeneity in viral gene expression in influenza-infected cells (67). Future studies of coronavirus gene expression dynamics may be particularly relevant for comparing viral gene expression between different cell types, coronaviruses, or between SARS-CoV-2 variants of interest, which have been described to have different kinetics of sgmRNA expression (70).

We demonstrated that 10X 5′ library preparation with extended R1 sequencing can be used to examine differential junction site usage within single cells (Fig. S6). Several groups have identified TRS-independent SARS-CoV-2 sgmRNAs (12, 13, 15), the functional significance of which remains unknown. It is possible that changes in junction site usage between cell types or during the course of infection may play a role in pathogenesis.

One technical advantage of 10X 5′ extended R1 sequencing and scCoVseq data processing is that neither requires modification of “out of the box” 10X library preparation steps. They therefore do not require technical optimization and are readily available to other researchers. We anticipate that established alternative “pseudoalignment” scRNA-Seq read assignment quantification workflows, such as kallisto/bustools (71), would also exhibit increased sgmRNA detection with the 10X 5′ extended R1 sequencing strategy, as additional coverage of leader-sgmRNA junction sites would improve pseudoalignment to otherwise redundant viral transcripts. Furthermore, both 10X 5′ extended R1 sequencing and scCoVseq data processing would be expected to be effective for scRNA-Seq studies of additional coronaviruses or arteriviruses, which generate leader sequence-containing sgmRNAs via discontinuous transcription. Similar sequencing strategies utilizing extended R1 to capture 5′ transcript start sites and thereby resolve other types of “nested” RNAs without leader sequences could be similarly effective for other virus families that generate such RNAs via alternative mechanisms.

It should be noted that there are limitations to our study. With the data set analyzed, we are unable to know the “ground truth” infection status of a cell processed for scRNA-Seq, and therefore we cannot assess the true accuracy of infection classification. An additional limitation of our data processing strategy is that quantification of viral genes with scCoVseq is dependent on accurate annotation of viral RNAs. We derived our annotation based on published empirically defined TRS-dependent RNAs (12), but this does not preclude the existence of other viral RNAs at other time points or other cell types not examined here. Importantly, we explicitly exclude TRS-independent RNAs from our analyses. Methods such as STARsolo (60) or sequencing 10X libraries with long read (e.g., PacBio) sequencing (67) may allow for the detection and quantification of viral RNAs without reference annotation and irrespective of TRSs.

## Data Availability

Raw and processed scRNA-Seq data are available at NCBI GEO (accession GSE189900) and the analysis code is available at GitHub.

## SUPPLEMENTAL MATERIAL

Figure S1 spectrum.00776-23-s0001.pdf Supplemental Figure 1.

Figure S2 spectrum.00776-23-s0002.pdf Supplemental Figure 2.

Figure S3 spectrum.00776-23-s0003.pdf Supplemental Figure 3.

Figure S4 spectrum.00776-23-s0004.pdf Supplemental Figure 4.

Figure S5 spectrum.00776-23-s0005.pdf Supplemental Figure 5.

Figure S6 spectrum.00776-23-s0006.pdf Supplemental Figure 6.

Table S1 spectrum.00776-23-s0007.docx Supplemental Table S1: scRNA-Seq filtering thresholds.

ASM does not own the copyrights to Supplemental Material that may be linked to, or accessed through, an article. The authors have granted ASM a non-exclusive, world-wide license to publish the Supplemental Material files. Please contact the corresponding author directly for reuse.
