# Supplementary material for: Unambiguous detection of SARS-CoV-2 subgenomic mRNAs with single-cell RNA sequencing
Source: Microbiol Spectr. 2023 Sep 7;11(5):e00776-23. doi: 10.1128/spectrum.00776-23 (PMC10580996; doi:10.1128/spectrum.00776-23)
Supplement: Figure S1 — Supplemental Figure 1. [file spectrum.00776-23-s0001.pdf]

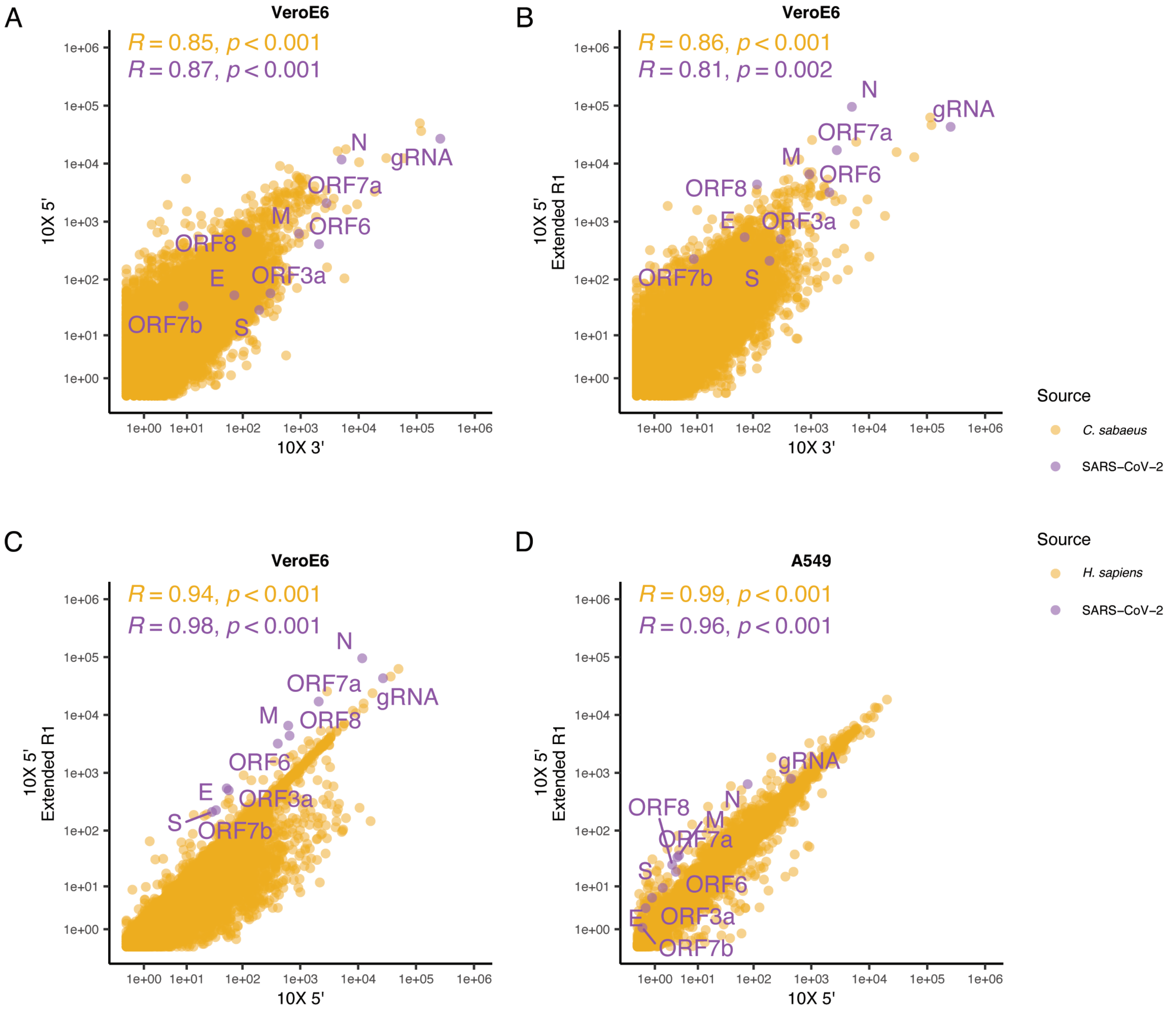

Supplemental Figure 1. Host (orange) and virus (purple) gene expression in Vero E6 cells (**A-C**) and ACE2-A549 cells (**D**) measured by 10X 3′, 10X 5′, and 10X 5′ with extended R1 sequencing. Each point represents the aggregate pseudobulk UMI count for a single gene assayed in the indicated assays per 10^6 total pseudobulk UMIs. Pearson correlation coefficients and resulting p values are indicated in each plot.
