# Supplementary material for: Unambiguous detection of SARS-CoV-2 subgenomic mRNAs with single-cell RNA sequencing
Source: Microbiol Spectr. 2023 Sep 7;11(5):e00776-23. doi: 10.1128/spectrum.00776-23 (PMC10580996; doi:10.1128/spectrum.00776-23)
Supplement: Figure S2 — Supplemental Figure 2. [file spectrum.00776-23-s0002.pdf]

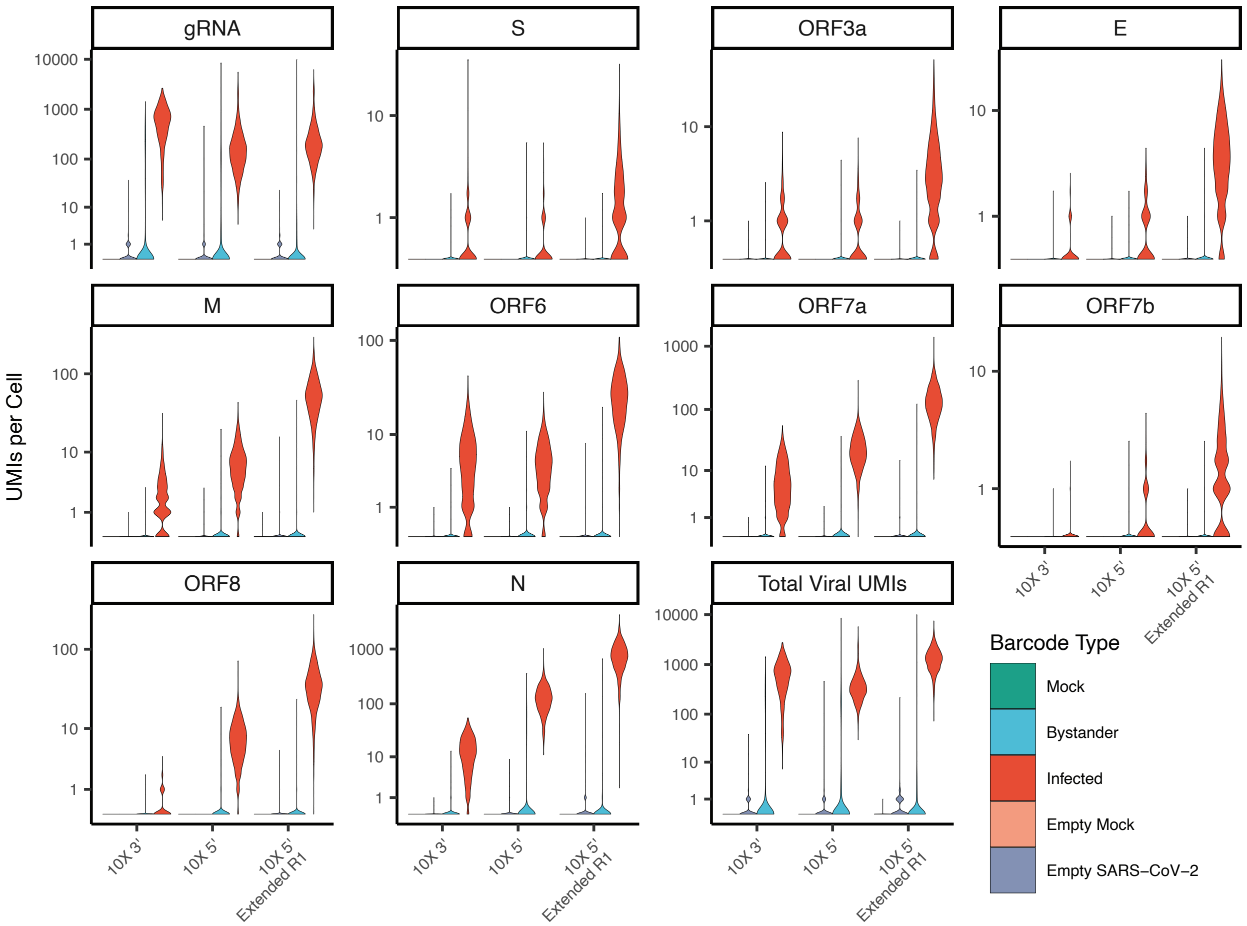

Supplemental Figure 2. SARS-CoV-2 (USA-WA1/2020) viral gene expression of infected and bystander Vero E6 cells and putative empty droplets from the mock and SARS-CoV-2 treated sample.
