# Supplementary material for: Unambiguous detection of SARS-CoV-2 subgenomic mRNAs with single-cell RNA sequencing
Source: Microbiol Spectr. 2023 Sep 7;11(5):e00776-23. doi: 10.1128/spectrum.00776-23 (PMC10580996; doi:10.1128/spectrum.00776-23)
Supplement: Figure S3 — Supplemental Figure 3. [file spectrum.00776-23-s0003.pdf]

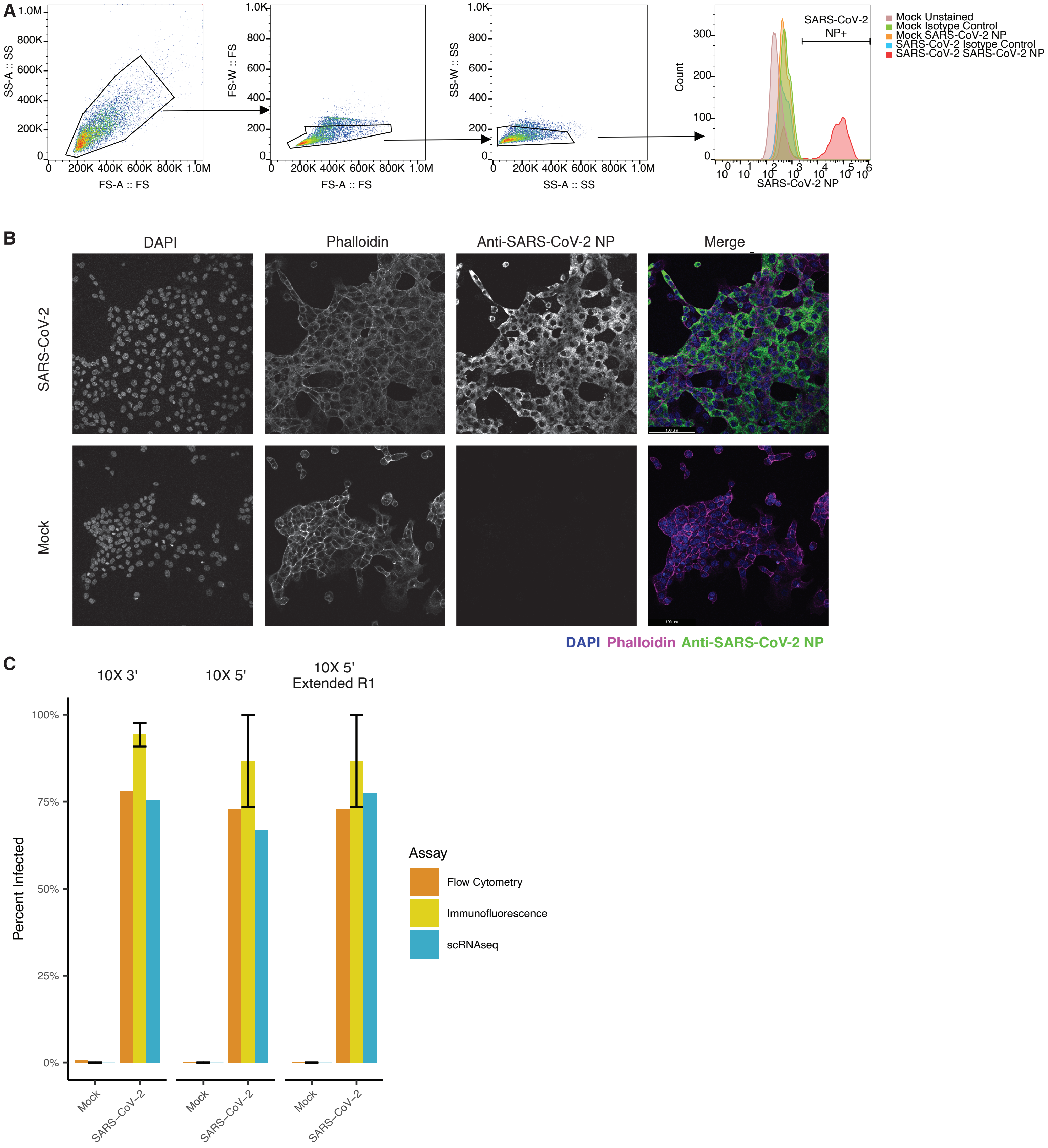

Supplemental Figure 3. **A**. Example flow cytometry plots illustrating gating strategy and SARS-CoV-2 NP labeling intensities in infected and control samples. **B**. Representative microscopy images of SARS-CoV-2 NP labeling for infected and mock cells. **C**. Percent of infected cells per sample as measured by flow cytometry, immunofluorescence, and scRNA-Seq. Because the same sample was sequenced with 10X 5′ and 10X 5′ extended R1, flow cytometry and immunofluorescence results are duplicated for ease of visualization. Error bars for immunofluorescence indicate mean ± one standard deviation of percent infected cells based on three fields per sample. All data from Vero E6 cells infected with SARS-CoV-2 (USA-WA1/2020).
