# Supplementary material for: Unambiguous detection of SARS-CoV-2 subgenomic mRNAs with single-cell RNA sequencing
Source: Microbiol Spectr. 2023 Sep 7;11(5):e00776-23. doi: 10.1128/spectrum.00776-23 (PMC10580996; doi:10.1128/spectrum.00776-23)
Supplement: Figure S4 — Supplemental Figure 4. [file spectrum.00776-23-s0004.pdf]

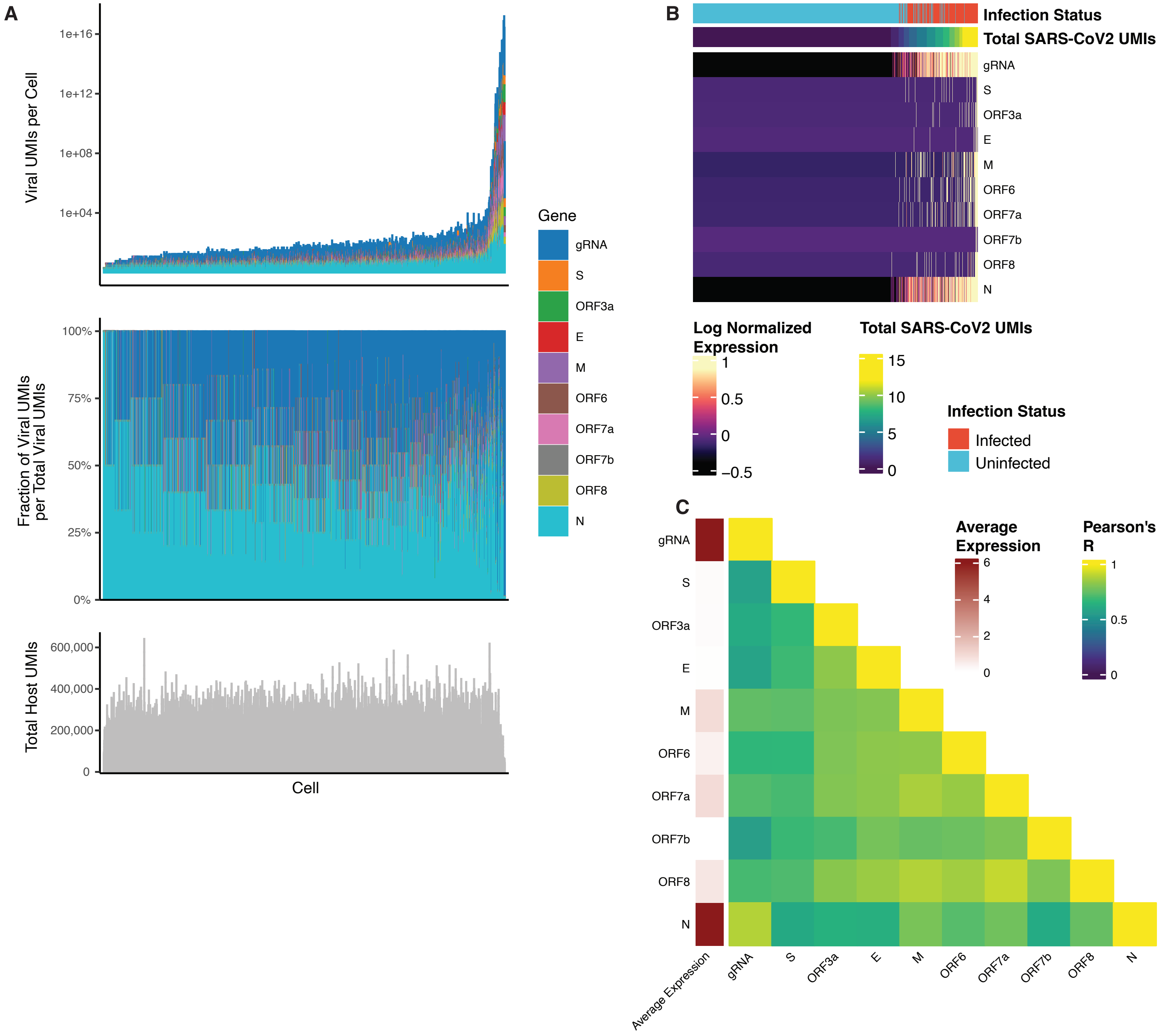

Supplemental Figure 4. Expression of rSARS-CoV-2 ORF6 M58R viral genes in infected and uninfected ACE2-A549 cells. **A.** Top: Total viral UMIs per cell are plotted with each cell representing a single bar and cells ordered from left to right according to increasing total number of viral UMIs. The number of viral UMIs contributed by each viral gene is indicated by color. Middle: The fraction of viral UMIs derived from each viral gene scaled to 100% is shown with the color of each bar indicating the viral gene. Bottom: The total number of host UMIs detected per cell. **B.** Expression of viral genes in infected and uninfected ACE2-A549 E6 cells. Each column corresponds to a single cell and expression of each viral gene is indicated by color. Cells are ordered left to right by increasing total SARS-CoV-2 viral UMI counts. Cells with total SARS-CoV-2 viral UMIs greater than the 99th percentile are "clipped" to the 95th percentile value for improved visibility. Cells are annotated above with assigned infection status. **C.** Correlation of rSARS-CoV-2 ORF6 M58R viral gene expression across ACE2-A549 cells as measured in datasets generated by 10X 5′ extended R1 sequencing and processed with scCoVSeq. Sidebar annotation indicates average sgmRNA expression detected by each sequencing method.
