# Supplementary figures and images for: Unambiguous detection of SARS-CoV-2 subgenomic mRNAs with single-cell RNA sequencing
Source: Microbiol Spectr. 2023 Sep 7;11(5):e00776-23. doi: 10.1128/spectrum.00776-23 (PMC10580996; doi:10.1128/spectrum.00776-23)

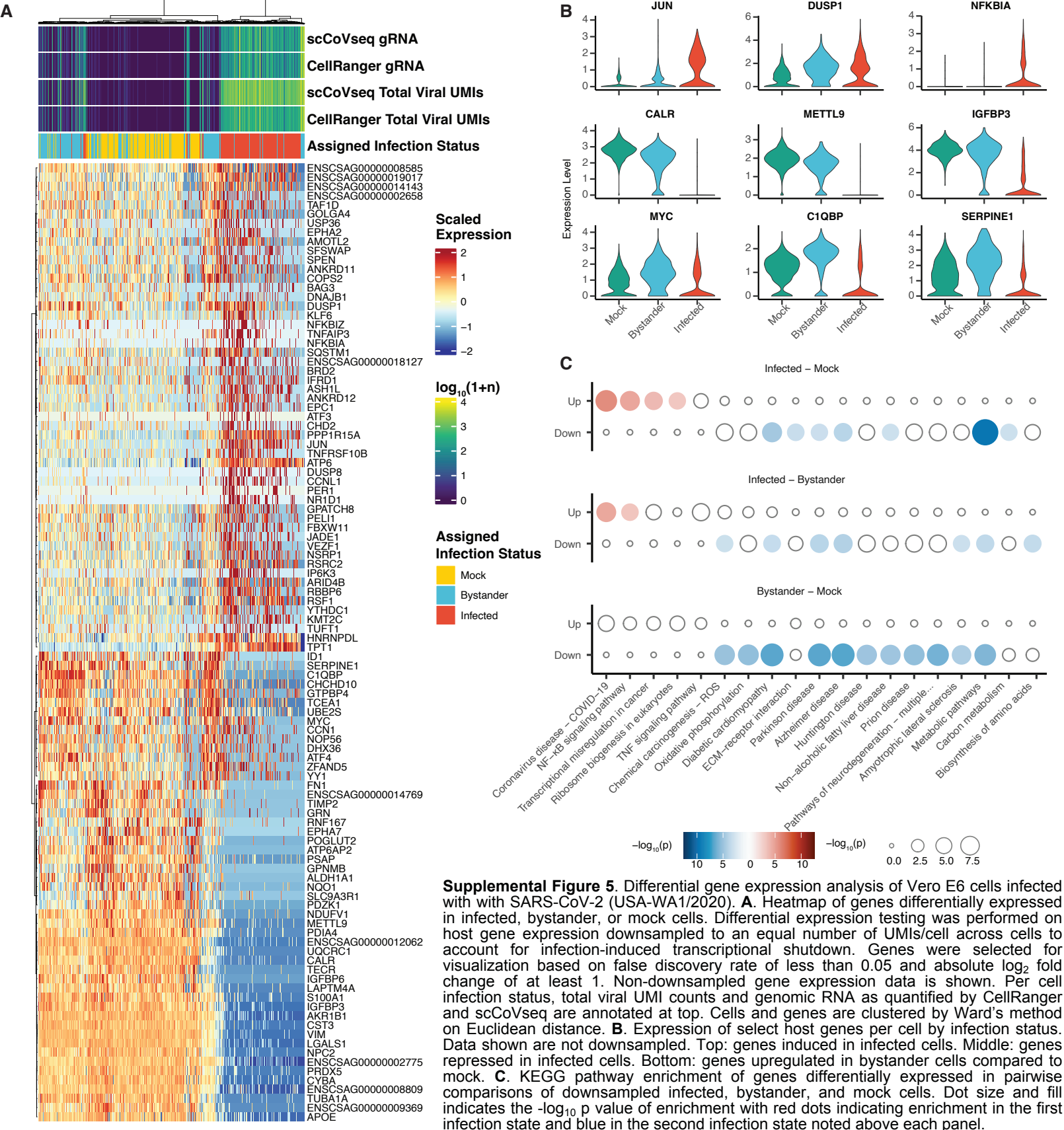

Supplement: Figure S5 — Supplemental Figure 5. [file spectrum.00776-23-s0005.pdf]
