# Supplementary material for: Unambiguous detection of SARS-CoV-2 subgenomic mRNAs with single-cell RNA sequencing
Source: Microbiol Spectr. 2023 Sep 7;11(5):e00776-23. doi: 10.1128/spectrum.00776-23 (PMC10580996; doi:10.1128/spectrum.00776-23)
Supplement: Figure S6 — Supplemental Figure 6. [file spectrum.00776-23-s0006.pdf]

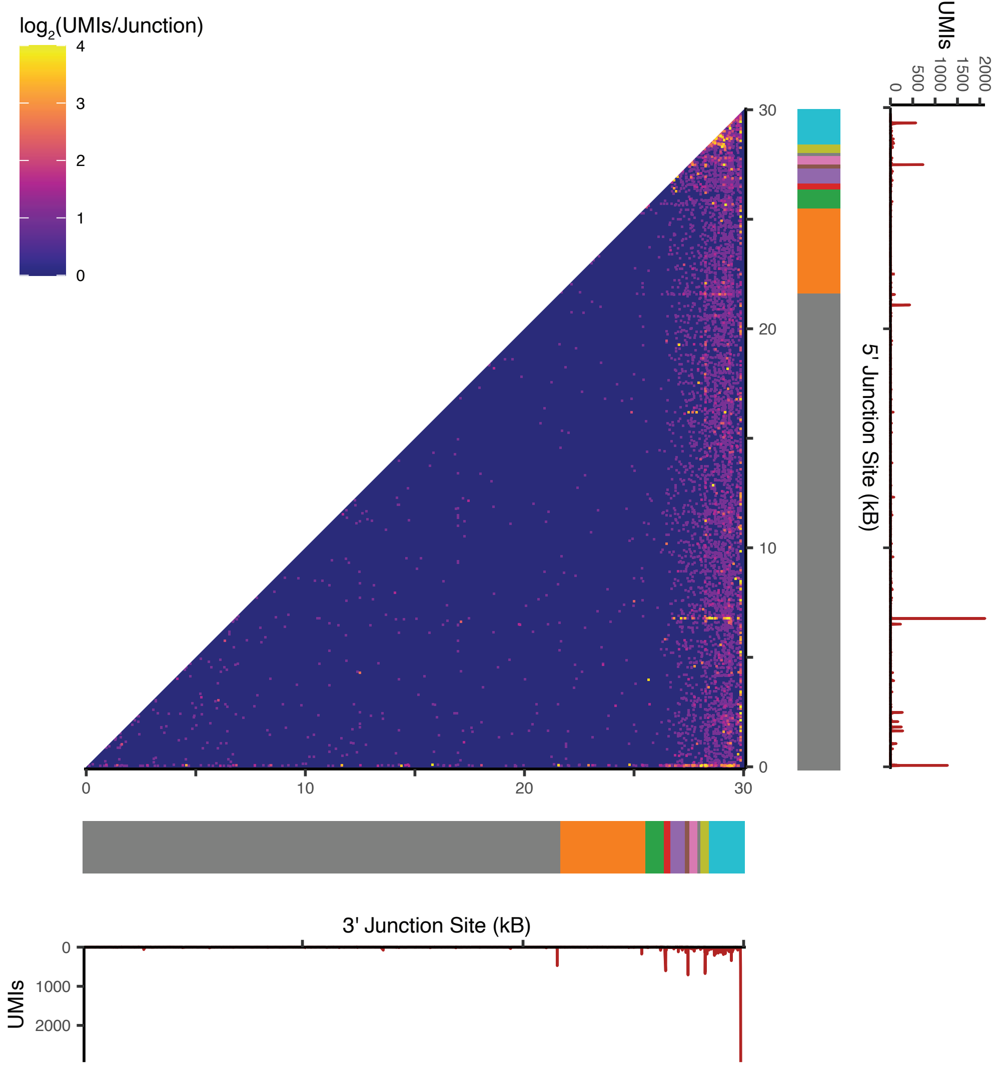

Supplemental Figure 6. Detection of junction sites in SARS-CoV-2 reads with 10X 5′ extended R1 sequencing in Vero E6 cells infected with with SARS-CoV-2 (USA-WA1/2020). Junction sites are represented by the 5′ start site and 3′ end site on the y- and x-axis, respectively. Color indicates the log2 total UMI count per junction across all cells in the SARS-CoV-2 infected sample. Below each axis, the number of UMIs supporting a position as a junction start or end site is indicated by density plot.
