# Supplementary material for: Unambiguous detection of SARS-CoV-2 subgenomic mRNAs with single-cell RNA sequencing
Source: Microbiol Spectr. 2023 Sep 7;11(5):e00776-23. doi: 10.1128/spectrum.00776-23 (PMC10580996; doi:10.1128/spectrum.00776-23)
Supplement: Table S1 — Supplemental Table S1: scRNA-Seq filtering thresholds. [file spectrum.00776-23-s0007.docx]

| **Sample** | **Total UMIs/cell** | **Detected genes/cell** | **% mitochondrial gene expression** |
| --- | --- | --- | --- |
|  |  |  |  |
| **Vero E6** | | | |
| 10X 3′ mock | $\geq$3,000 | 400 - 4,000 | $\leq$2.25% |
| 10X 3′ infected | NA | 10 - 3,000 | $\leq$3.0% |
| 10X 5′ mock | $\geq$3,000 | NA | $\leq$0.75% |
| 10X 5′ infected | $\geq$3,000 | 10 - 7,000 | $\leq$0.75% |
| 10X 5′ extended R1 mock | $\geq$3,000 | $\leq$6,000 | $\leq$1.0% |
| 10X 5′ extended R1 infected | 200 - 4,500 | 10 - 5,500 | $\leq$1.0% |
|  |  |  |  |
| **A549-ACE2** | | | |
| 10X 5′/5′ extended R1 mock | $\geq$5,000 | $\geq$1,000 | $\leq$10% |
| 10X 5′/5′ extended R1 M58R infected | $\geq$5,000 | $\geq$1,000 | $\leq$10% |

**Supplemental Table S1: scRNA-Seq filtering thresholds**. For each sample, cells meeting the listed thresholds were included in downstream analyses.
